# Design and Installed Performance Analysis of a Miniaturized All-GNSS Bands Antenna Array for Robust Navigation on UAV Platforms

**DOI:** 10.3390/s22249645

**Published:** 2022-12-09

**Authors:** Simon P. Hehenberger, Wahid Elmarissi, Stefano Caizzone

**Affiliations:** Institute of Communication and Navigation, German Aerospace Center (DLR), 82234 Wessling, Germany

**Keywords:** UAV, navigation, GNSS, antenna array, installed performance, antenna, robust navigation

## Abstract

Global navigation satellite systems (GNSS) are vital technologies of our age and serve a plethora of industries that rely on precise positioning for automation, efficiency, and safety. Emerging applications of unmanned aerial vehicles (UAV) in critical applications like security, surveillance, critical logistics and defense demand precise and robust navigation capabilities even in challenging environments with high multipath or (un-)intended interference. The design of robust GNSS receivers for UAV applications, capable of suppressing interfering signals, is challenging due to the need for multi-antenna systems and the stringent requirements on hardware to be lightweight and miniaturized enough to fit onto small mobile platforms. In order to overcome these limitations, the present article details a four-element wideband antenna array, fitting into a 100 mm diameter footprint. The array is capable to operate across all GNSS frequency bands while incorporating, if needed, a multipath mitigation solution. The antenna design relies on a modular concept with 3D printed Dielectric Resonator Antennas (DRAs) and vertical choke rings. The antenna performance is evaluated in terms of its radiation pattern via installed antenna simulations and measurements in an anechoic chamber. The effect of different installation heights on the antenna pattern is studied. Furthermore, GNSS measurements carried out with the array alone and mounted on the UAV are presented.

## 1. Introduction

There is an increasing trend to utilize UAVs for applications like security and surveillance as well as critical logistics. However, UAVs carrying out sensitive tasks require not only good path planning [1], but also precise and robust positioning to ensure efficient and safe operation in challenging environments with (un-) intentional interference and strong multipath phenomena. Currently the most common solution for precise and drift-free positioning is the use of GNSS systems, thanks to several attractive features: global coverage, lightweight and high accuracy. However, GNSS systems are prone to jamming and spoofing [2,3]. There has been increased awareness of the fact that current state of the art mobile navigation equipment is insufficiently equipped to operate in GNSS-challenged or denied environments [4].

Commonly employed mobile satellite navigation receivers are vulnerable against interference and jamming (both intentional and unintentional) due to the low signal power at the receiver and the commonly employed single-channel receiver architectures. This vulnerability has driven the development of innovative multi-antenna and receiver solutions [5,6] that are capable of suppressing interferences from specific directions by introducing null steering in the combined antenna pattern.

A further drawback of GNSS equipment for autonomous UAV applications is the dependence of the achievable accuracy on the environment due to multipath propagation of the satellite signals. For example, the multipath effect in urban environments severely handicaps the achievable positioning performance. GNSS antennas generally are supposed to receive signals in RHCP polarization and suppress all signals in LHCP polarization in order to be resilient against first-order multipath signals. The most prominent contribution to LHCP radiation is usually the antenna’s back lobe and GNSS antennas for ground stations commonly employ horizontal choke rings to mitigate this back-lobe contribution and increase accuracy. However, these structures are bulky and heavy and therefore not suitable for mobile, small-body platforms like UAVs. In a recent development, a novel choke-ring design for LHCP back lobe suppression built from vertical layers was introduced [7]. Furthermore, in [8] it has been shown through a simulation study that this vertical choke ring structure has great potential for application on UAV platforms.

Besides the challenges posed by (un-) intentional interference and jamming as well as strong multipath environments, it is critical that GNSS systems, employed on compact UAV platforms, are designed to be lightweight, low-power and sufficiently miniaturized. Significant effort has been invested to miniaturize GNSS antenna systems [9,10] in order to be compliant with existing dimensional regulations for GNSS systems in automotive and aeronautical industry standards. This work builds on the miniaturized wideband GNSS antenna array introduced in [8] and discusses numerical simulations as well as experimental verification of the antenna array performance. The standalone as well as the installed performance of the antenna array on a UAV is investigated by numerical simulations. Furthermore, measurements to study the effect of different installation heights and vertical choke ring configurations of the antenna on the UAV are carried out. Furthermore, GNSS field measurements with the standalone antenna-array are compared to measurements of the GNSS performance on the UAV.

This article is structured as follows. In Section 2, the antenna concept for precise and robust navigation on small mobile platforms is discussed in detail, giving insights into the manufacturing and performance of the individual antennas and the array. Section 3 deals with the installed performance of the antenna on the intended UAV. The installation effects are estimated first via electromagnetic numerical solvers and later compared to measurements of the antenna performance on the UAV. GNSS field measurements are presented in Section 4. A discussion and outlook on future research work will be given in Section 5.

## 2. Antenna Array

### 2.1. Antenna Concept

**Figure 1:**
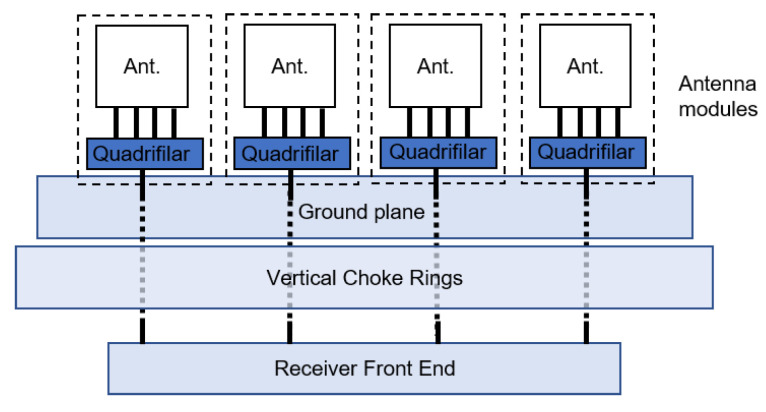
Concept of the miniaturized antenna array for UAV applications consisting of individual antenna modules mounted on a ground plane and connected to the receiver frontend.

The antenna concept, as depicted in Figure 1, consists of a 2 × 2 array, built from individual wideband dielectric resonator antenna (DRA) modules with a quadrifilar feed. The array can be mounted on top of a choke ring structure tailored to suppress UAV-platform-generated multipath in the L1/E1 (central frequency = 1575 MHz) and L5/E5a band (central frequency = 1176 MHz). The antenna module outputs are then further processed in an ad-hoc front end developed by project partners and not dealt with in this paper. 

### 2.2. Antenna Module

**Figure 2:**
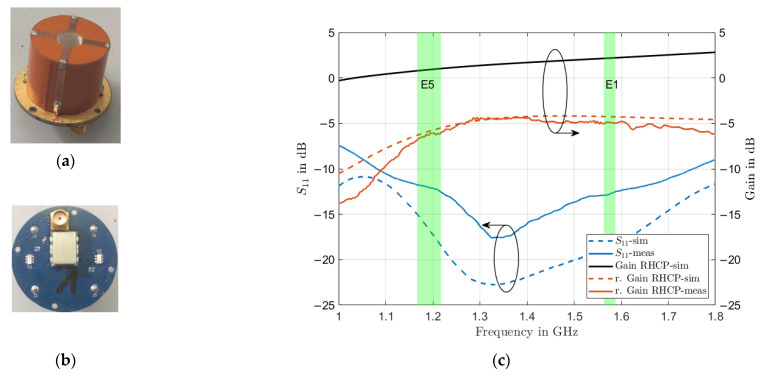
Wideband annular cylindrical dielectric resonator antenna module. (**a**) Resonator body with conductive conformal feed-strips glued on a circular piece of PCB, acting as ground plane; (**b**) Quadrifilar feed-network on the backside of the PCB with SMA connector and soldered pins to connect to the feed-strips of the DRA; (**c**) Comparison of simulated and measured S-parameters and realized RHCP gain values at the zenith of the antenna module.

The individual antenna modules, as depicted in Figure 2a, consist of an annular dielectric cylinder with a diameter of 30 mm and height of 25 mm with four conformal conductive feed strips as introduced in [11]. The cylinder is glued to a 1.6 mm thick circular printed circuit board (PCB) (Figure 2b), which inhabits a quadrifilar circuit, responsible for creating the necessary 90° phase shifts in the four individual feed points of the antenna to achieve right-hand-circular-polarization (RHCP). The glue (3M Adhesive 465) has a permittivity of 2.5 and a height of 0.1 mm. The cylinder is additively manufactured via a fused deposition modeling process, employing the PREPERM ABS1000 filament with a permittivity of $\varepsilon_{r}=10$ and a loss tangent of tanδ=0.003, as reported in its respective datasheet [12]. A known peculiarity of additive manufacturing is that extrusion parameters have a significant influence on the effective material parameters of the resulting material [13,14]. Therefore, a material characterization has been carried out, as reported in [11]: the printing process resulted in reducing the effective permittivity of the substrate to $\varepsilon_{r}=8.2$. Another commonly encountered challenge when dealing with dielectric resonator antennas is the selective metallization of areas in order to create conformal feed and matching structures. This problem has been successfully addressed in [8] by utilizing metallized Kapton polyimide sheets wrapped around the DRA, allowing precise positioning of the feed strips relative to each other. Simulated and measured S-parameters, as well as RHCP realized gain values at broadside (θ=0), are compared in Figure 2c. The antenna module achieves an input reflection coefficient of <−10 dB over all GNSS frequency bands and a maximum realized RHCP gain of −4.5 dB at zenith. While the gain of the antenna is 1 dB and 2.5 dB at L5/E5a and L1/E1, respectively, the realized gain of the antenna is relatively low. The reason for the reduced realized gain is the inter-mode coupling in the quadrifilar feed of the antenna (where a significant part of the power is reabsorbed by the opposite feed-strip and therefore not radiated). Furthermore, there are additional losses caused by the 180° hybrid and two 90° hybrids used to create the wideband 90° phase shifts in the four individual feeds. Although the quadrifilar feed approach results in lower realized gain, the benefit is lower cross-polarization levels, as well as a more stable phase center, group delay, and axial-ratio behavior of the antenna, which overall improve the GNSS performance of the antenna [15]. Furthermore, the quad-feed approach helps to keep the mutual coupling of antenna elements embedded in the array configuration at acceptable levels, as discussed in the next section. Due to the later employed larger ground plane and array configuration, the realized gain will still be increased to adequately receive GNSS signals. 

### 2.3. Array

**Figure 3:**
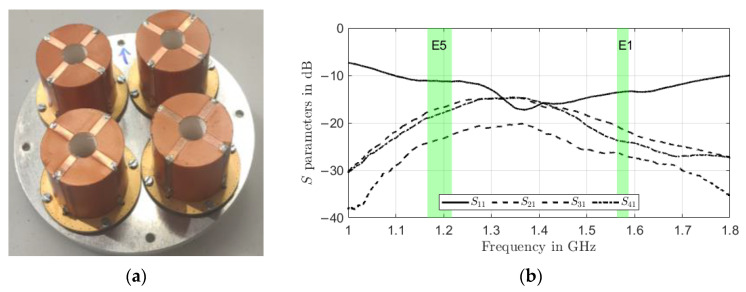
Miniaturized wideband GNSS antenna array. (**a**) Array configuration from individual annular ring dielectric resonator antennas in 2 × 2 quadratic configuration; (**b**) Measurement of scattering parameters between array elements showing a reflection coefficient below −10 dB for the embedded element and a maximum mutual coupling of −15 dB between neighboring array elements.

Individual antenna modules are mounted on an aluminum ground plane with 100 mm diameter in a 2 × 2 quadratic configuration, as depicted in Figure 3a. The array, with an inter-element spacing of 40 mm or approximately 0.16 and 0.21 wavelengths at E5a/L5 and L1/E1 center frequencies respectively, is strongly miniaturized. The reflection coefficient and coupling of antenna module 1 in the array as a function of frequency is plotted in Figure 3b.

**Figure 4:**
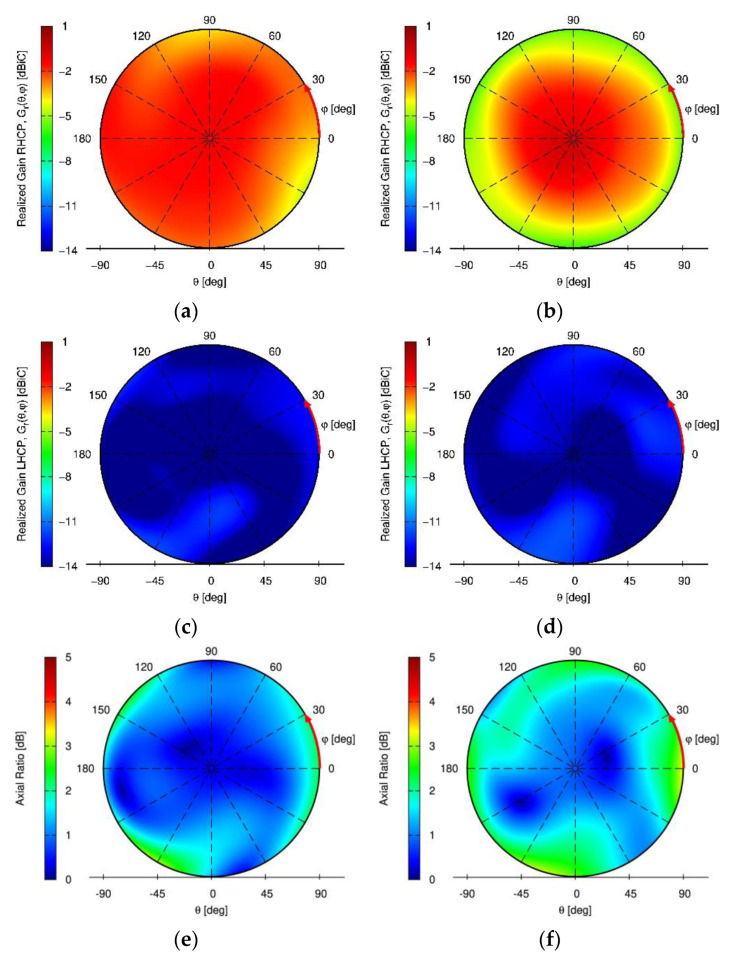
Measured miniaturized wideband GNSS antenna array measurement results of the array-mode radiation pattern. (**a**) Right hand circular polarization (RHCP) realized gain at L5/E5a; (**b**) RHCP realized gain at L1/E1; (**c**) left hand circular polarization (LHCP) realized gain at L5/E5a; (**d**) LHCP realized gain at L1/E1; (**e**) axial ratio (AR) at L5/E5a; (**f**) AR at L1/E1.

Besides a satisfying S11 of <−10 dB of the embedded elements over all GNSS frequency bands achieved, the maximum coupling between neighboring elements is below −15 dB, which is an excellent performance for an array with such strong miniaturization (and without using additional and bulky decoupling networks). Low mutual coupling is especially important for null-steering applications, as a high mutual coupling would reduce the available degrees of freedom for null-steering in signal processing. The measured RHCP and LHCP realized gain pattern and axial ratio pattern (AR) of the antenna in array mode (i.e. when all elements are excited uniformly) in the upper hemisphere (θ∈[0° 90°], φ∈[0° 360°]) are shown for the L5/E5a and L1/E1 center frequencies, respectively, in Figure 4. A maximum realized gain of about -1dBi is observed in Figure 4a,b for the L1/E1 and L5/E5a band.

It is worth highlighting that the combiner used for achieving array mode introduces about 2 dB of additional losses: in real-life use, where all antenna outputs are directly fed into the frontend channels and then combined digitally, such losses would not be present and therefore a gain above 0dBi would be achieved.

Excellent axial ratio values of below 3 dB over the whole upper hemisphere are achieved in both the L5/E5a and L1/E1 frequency bands (Figure 4c,d).

### 2.4. Vertical Choke Rings

**Figure 5:**
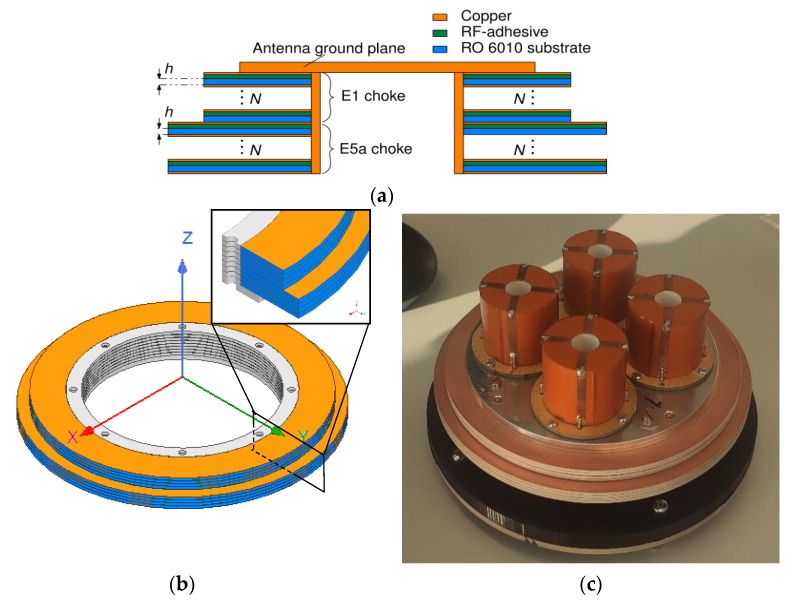
Miniaturized vertical choke rings for multipath mitigation. (**a**) Schematic; (**b**) Isometric view of the choke rings; (**c**) Manufactured vertical choke rings with antenna array.

GNSS antennas generally are supposed to receive signals in RHCP polarization and suppress all signals in LHCP polarization in order to be resilient against multipath signals originating from the ground or nearby objects. Multipath is known to be one of the most important error sources in precise GNSS receivers; its characterization and suppression is of paramount importance to achieve precise navigation [16]. A suitable method for LHCP suppression at discrete frequencies on small mobile platforms was introduced in [7] via the utilization of cavities parallel to the antenna ground plane. This concept was further developed in [8] via a simulation study which investigated the potential for miniaturization via introducing a dielectric in said vertical cavities. This work makes use of the investigation presented in [8], and a vertical choke ring combination with N = 4 rings based on h = 1.27 mm thick Rogers RO6010 substrate was chosen for manufacturing. A schematic of the vertical choke ring structure as well as a picture of the assembled antenna with choke rings are depicted in Figure 5. The inner wall of the vertical choke structure is made from metal rings that are press-fit into the individual Rogers RO6010 rings. The individual pieces are stacked onto a bottom ring with a larger outer diameter and are pressed together tightly by screws when the array is mounted on top. This configuration allows a compact and flexible arrangement of the antenna system.

The impact of the vertical choke ring structure on the GNSS performance is evaluated in the following section about installed performance. 

## 3. Installed Performance

The installation platform, in this case a hex rotor UAV, might cause large multipath contributions that negatively affect the positioning accuracy [16]. Furthermore, precise knowledge of the installed antenna pattern is beneficial to improve null-steering performance for interference suppression [17].

It is therefore necessary to properly characterize the antenna performance once installed on the envisaged UAV. This can be done both in a simulative and in a measurement-based manner.

Although an installed performance analysis via numerical simulations is simpler and less expensive and allows to evaluate multiple scenarios (e.g., installation heights), this technique is limited by the accurate knowledge of the final platform. Moreover, it can only approximately consider some effects present on the real-life platform, for instance the effect of additional RF components present for communication as well as the actual materials used for the UAV structure. Therefore, in this work we will show both simulative and measurement-based results to accurately estimate the influence of the active UAV onto the GNSS performance. Moreover, the effects of the installation platform for different antenna installation heights will be analyzed.

### 3.1. Simulation of Installed Performance

**Figure 6:**
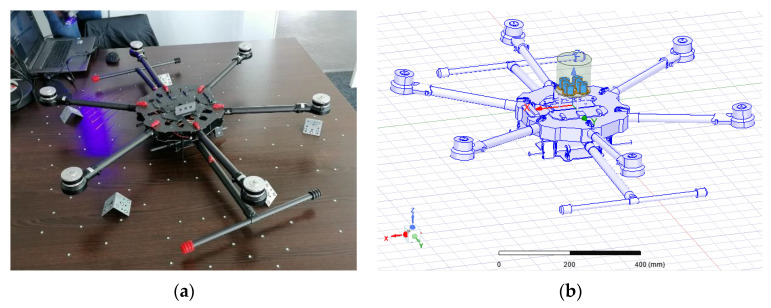
3D scan of hex rotor UAV. (**a**) Scanning process. (**b**) 3D digital model of the scanned hex rotor UAV, imported into Ansys High Frequency Structure Simulator, with finite element model of the antenna array.

In order to predict the influence of the installation platform onto the antenna performance, as described in [15], a sufficiently accurate model of the designated UAV is necessary. This was achieved via a 3D scan of the designated hex rotor UAV platform, performed by the company Creaform through their HandySCAN BLACK Elite, which delivered an accurate digital 3D representation, as depicted in Figure 6. 

The installed performance simulation of the antenna on the UAV is performed via a hybrid approach, where the antenna array is solved with a finite element method, enclosed with a finite element boundary condition, and the scattering from the platform is considered as an integral equation problem where the UAV is modeled to be perfectly conductive. The perfect conductive representation of the UAV is carried out to represent a worst-case scenario where every part of the UAV is highly reflective and thus has a strong interaction with surrounding electromagnetic fields. Furthermore, this perfectly conductive representation of the UAV results in reduced numerical complexity, as the fields inside the UAV body are zero and the scattering problem simplifies to a computation of surface currents. 

Multiple possible installation heights (i.e., distance between the antenna ground plane and top face of the UAV platform) are considered and evaluated in the range h = 50 mm to h = 130 mm.

**Figure 7:**
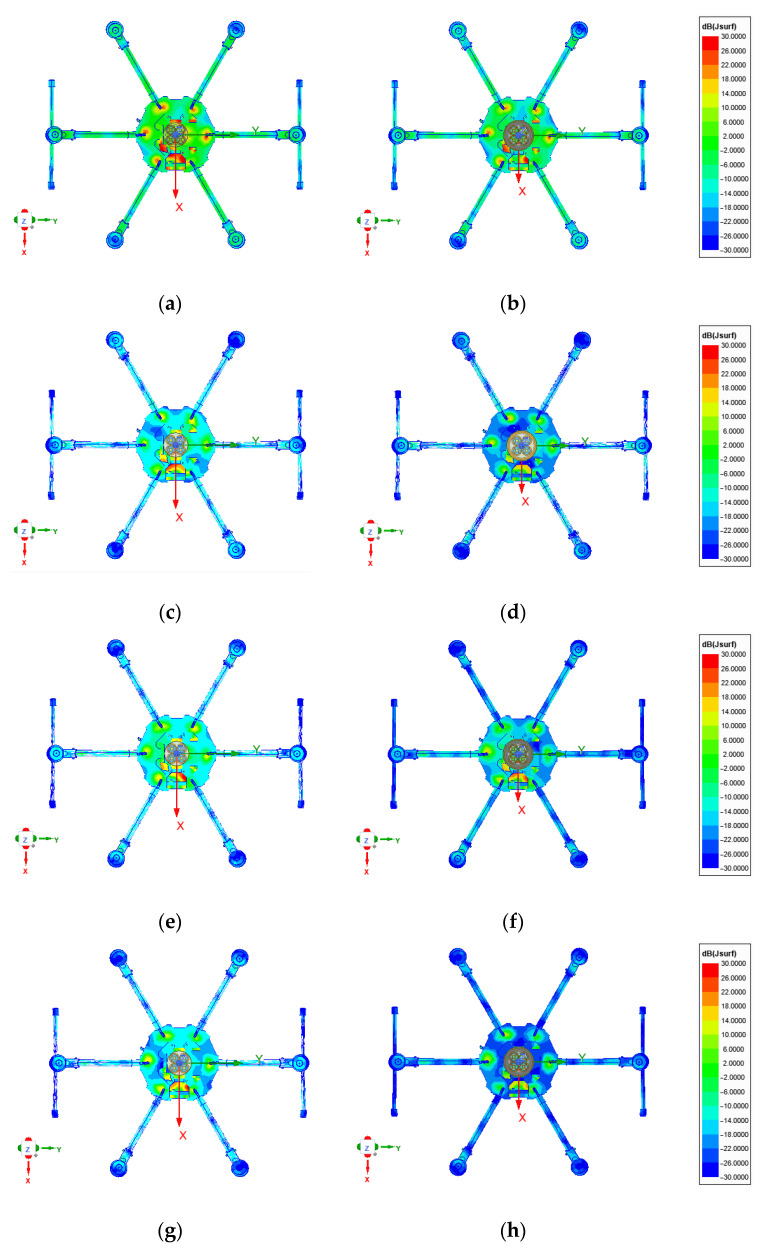
Simulated surface currents at the E5a and E1 center frequencies on a hex-rotor UAV for the miniaturized GNSS array without (left) and with (right) vertical choke rings and different installation heights. (**a**) L5/E5a, h = 50 mm wo. vertical choke; (**b**) L5/E5a, h = 50 mm w. vertical choke; (**c**) L5/E5a, h = 110 mm wo. vertical choke; (**d**) L5/E5a, h = 110 mm w. vertical choke; (**e**) L1/E1, h = 50 mm wo. vertical choke; (**f**) L1/E1, h = 50 mm w. vertical choke; (**g**) L1/E1, h = 110 mm wo. vertical choke; (**h**) L1/E1, h = 110mm w. vertical choke.

The simulated surface currents at the L5/E5a and L1/E1 center frequencies for installation heights h = 50 mm and h = 110 mm are depicted in Figure 7 for the antenna with and without vertical choke rings, respectively. 

The effects of choke rings and installation heights can already be qualitatively observed, as the magnitude of surface currents is reduced with increasing installation height and for simulations including the vertical choke rings. 

**Figure 8:**
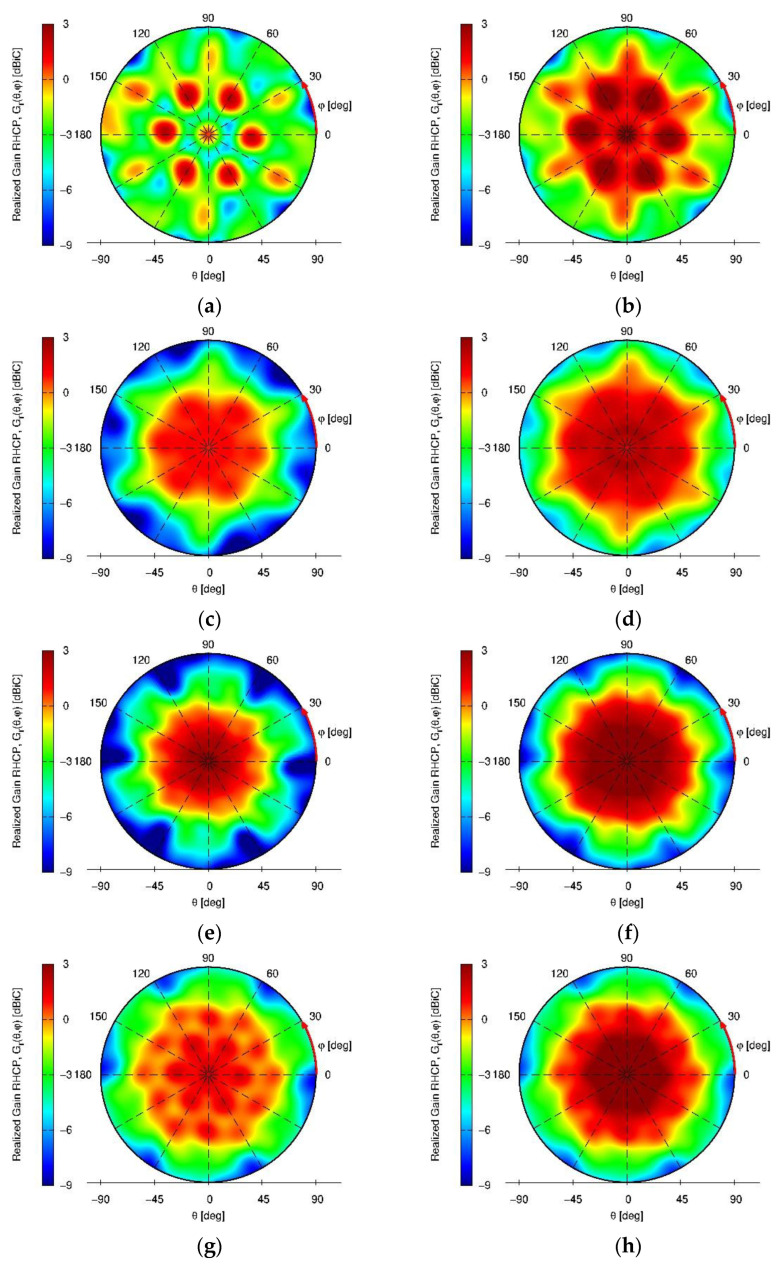
Simulated array RHCP realized gain patterns for L5/E5a and L1/E1 band center frequencies on a hex-rotor UAV for the miniaturized GNSS array with and without vertical choke rings and different installation heights. (**a**) L5/E5a, h = 50 mm wo. vertical choke; (**b**) L5/E5a, h = 50 mm w. vertical choke; (**c**) L5/E5a, h = 110 mm wo. vertical choke; (**d**) L5/E5a, h = 110 mm w. vertical choke; (**e**) L1/E1, h = 50 mm wo. vertical choke; (**f**) L1/E1, h = 50 mm w. vertical choke; (**g**) L1/E1, h = 110 mm wo. vertical choke; (**h**) L1/E1, h = 110 mm w. vertical choke.

The installation effects on the resulting RHCP realized gain patterns of the antenna with and without the vertical choke rings on the platform for installation heights 50 mm and 110 mm are depicted in Figure 8 for the center frequencies of the L5/E5a and L1/E1 band respectively.

**Figure 9:**
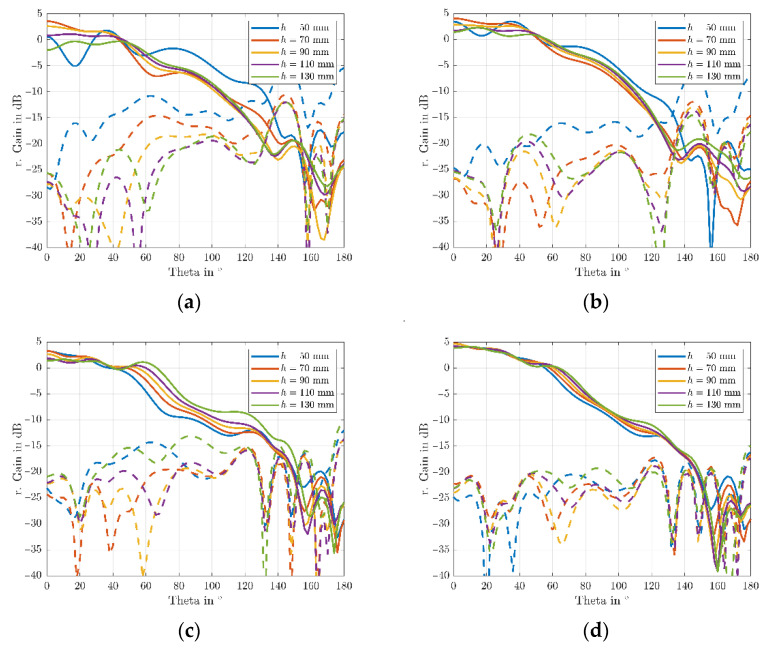
Simulated RHCP and LHCP realized gain patterns of the miniaturized GNSS array with and without vertical choke rings on a hex-rotor UAV for different installation heights. (**a**) L5/E5a without vertical choke; (**b**) L5/E5a with vertical choke; (**c**) L1/E1 without vertical choke; (**d**) L1/E1 with vertical choke.

Furthermore, 1D azimuth cuts of the array-mode RHCP and LHCP realized gain, with and without the vertical choke rings, at the L5/E5a and L1/E1 center frequencies (respectively 1176 MHz and 1575 MHz) are plotted in Figure 9 for a larger set of installation heights. 

It is observed, that the installation height of the antenna array above the UAV has a significant influence on the array performance. Low installation heights show severe ripples in the antenna array realized gain pattern and heightened back-lobe magnitudes. This effect is especially prominent in the L5/E5a band. Increasing installation height results in a smoother pattern in the upper hemisphere and reduced back-lobe magnitudes.

Furthermore, a clear improvement through the application of the vertical choke rings in both the L5/E5a and L1/E1 band, with patterns being more constant for different heights (i.e., enabling to obtain a better platform independency), can be observed. Although the simulation results are obtained in a worst-case scenario, due to the modelling of the UAV as perfectly electrically conductive, it gives insight into preferable mounting modalities. In case the antenna needs to be mounted very close to the platform (e.g., at h = 50 mm), choke rings would be strongly beneficial. On the other hand, if higher distances are feasible (from a mechanical and system-level point of view), the use of choke rings could be possibly omitted, contributing to minimizing weight and cost of the system. It is worth highlighting that the effects of the installation platform cause a significant rise of the crosspolarization (and hence of the axial ratio) for small heights, that the choke rings can only partially avoid. On the other hand, increased installation height shows to mitigate this degradation and enable to preserve better polarization purity. While the AR is below 3 dB for an angular range of θ<50° for an installation height of 50 mm, the angular range widens up to θ<80° when installed at a height of 110 mm above the UAV.

### 3.2. Measurement of Installed Performance

**Figure 10:**
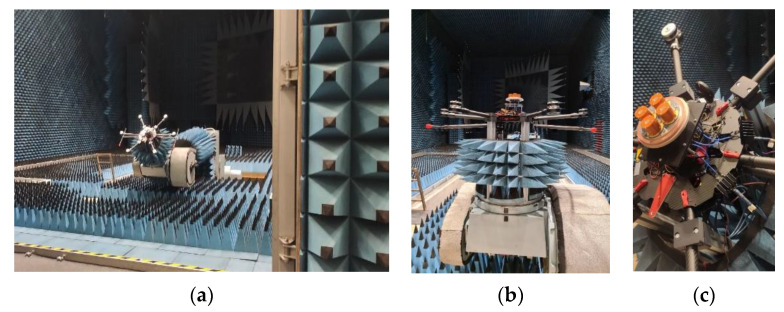
Installed performance measurement setup of the miniaturized GNSS antenna array on a hex-rotor UAV at the German Aerospace Center—Compact Test Range anechoic chamber. (**a**) View of the installed UAV in the measurement chamber; (**b**) Detailed view of UAV with antenna on the positioner; (**c**) Detailed view of the antenna with vertical choke rings installed on the UAV.

While the numerical simulations discussed above give valuable insight into choosing an installation configuration of the antenna on the UAV, it is still not completely representative of the real-world achievable performance, due to the simplifications done for simulation. To test the miniaturized GNSS array performance on its designated installation platform, installed performance measurements have been carried out in the Compact Test Range (CTR) of the German Aerospace Center. The antenna array installed performance has been measured with and without choke-ring, for the installation height of 110mm above the UAV base plate for comparison with simulation results. During the measurement, the UAV, without rotors, was mounted on a 6-axis positioner of the anechoic chamber, as depicted in Figure 10. 

**Figure 11:**
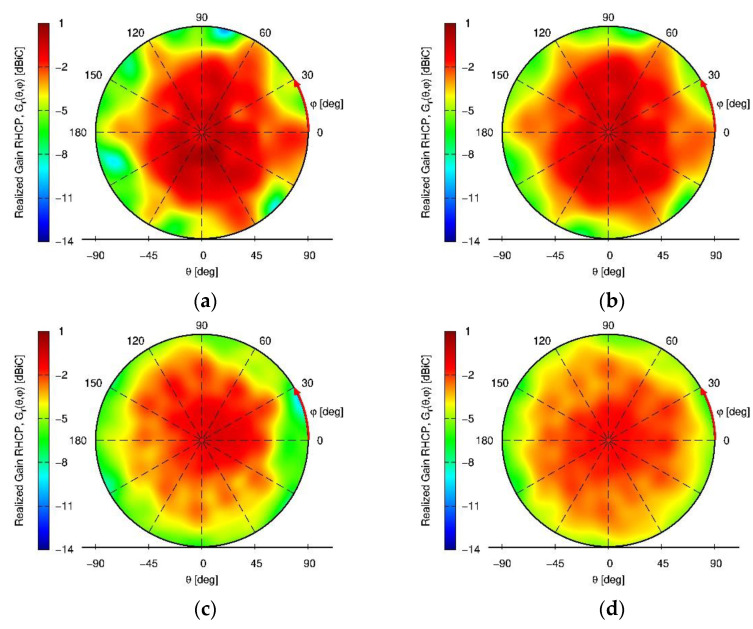
Measured RHCP and LHCP realized gain patterns of the miniaturized GNSS array with and without vertical choke rings on a hex-rotor UAV for an installation height of 110 mm. (**a**) L5/E5a, h = 110 mm wo. vertical choke; (**b**) L5/E5a, h = 110 mm w. vertical choke; (**c**) L1/E1, h = 110 mm wo. vertical choke; (**d**) L1/E1, h = 110 mm w. vertical choke.

The positioner and UAV holding structure was covered with absorbers to minimize their influence on the measurements. The signals of individual antenna modules were combined with the appropriate phase shift to measure the array radiation pattern. The results are depicted for the L1/E1 and L5/E5a center frequency respectively in Figure 11. For comparison with simulations the measured realized gain patterns color-scale in this figure is shifted by +5 dB in order to account for the losses in the individual antenna module quadrifilar feed networks and the signal phase shifter and power combiners used to measure the array pattern. 

As a matter of fact, the chosen installation height of 110 mm appears to be already enough to decouple the antenna from the UAV structure and therefore the improvements obtained with the choke ring in this case are not substantial for medium to high elevations (with better reception still to be seen for low elevations, where minima in the pattern are smoothed/less deep).

At the purpose of minimizing weight and complexity, it is therefore chosen to proceed at this height and with no choke rings for the GNSS validation of next session.

## 4. GNSS Measurements

**Figure 12:**
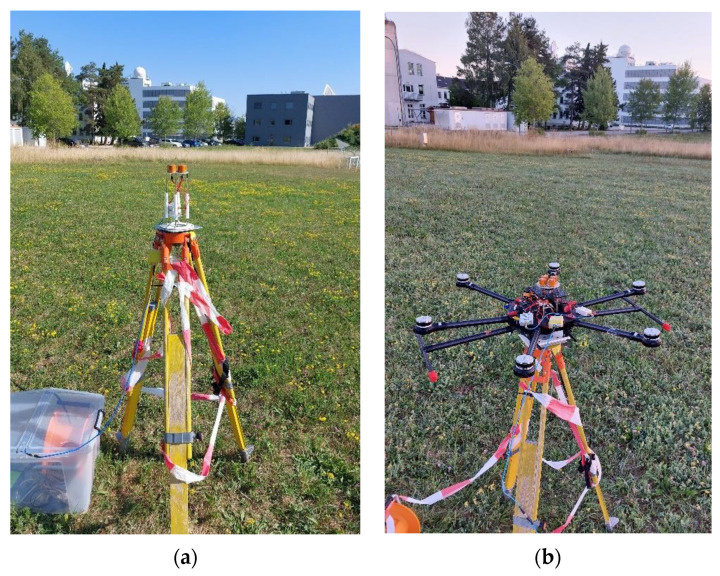
GNSS field measurements of the antenna array. (**a**) Standalone; (**b**) Antenna array mounted on UAV. Measurements were performed at exact same location and height above ground, only photographed from slightly different angles.

**Figure 13:**
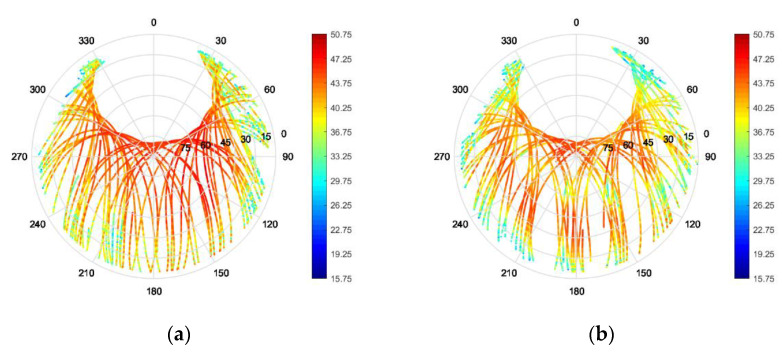
GNSS field measurements of the antenna array. Sky plot of C/N0 in dB of received GNSS signals at L1/E1 band over a 24-h time window. (**a**) Antenna array standalone; (**b**) Antenna array mounted on UAV.

**Figure 14:**
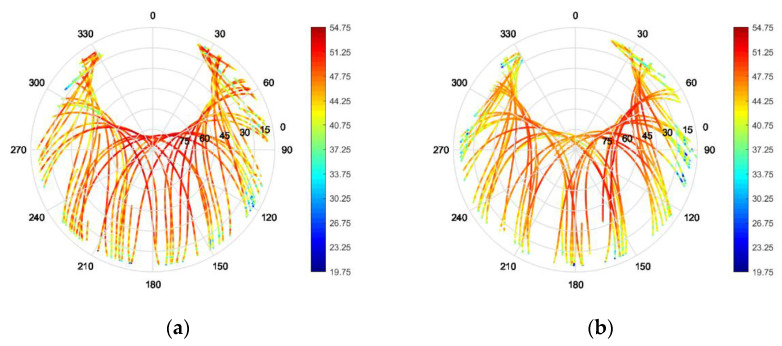
GNSS field measurements of the antenna array. Sky plot of C/N0 in dB of received GNSS signals at L5/E5a band over a 24-h time window. (**a**) Antenna array standalone; (**b**) Antenna array mounted on UAV.

Further verification of the antenna array beyond the idealized conditions of numerical simulations and antenna anechoic chambers has been carried out by performing GNSS field measurements. The measurement campaign was performed over the span of 24 h for both the standalone antenna array as well as the antenna array mounted on the UAV at an installation height of 110 mm and without choke rings, as discussed in previous Section and depicted in Figure 12. The measurement of both configurations was performed with the antenna at the same position and height above ground in an open field during rain-less, partly clouded 24 h time windows. The GNSS measurements were carried out with the antenna array connected to a Javad Delta receiver with an intermediate low noise amplifier. It is worth highlighting that GNSS measurement results are presented with a flipped elevation axis, as this is the standard in the GNSS community. Meaning the broadside of the array (in antenna patterns denoted as θ=0) corresponds to an elevation of 90° in GNSS measurements shown in Figure 13 and Figure 14 and following. 

The resulting satellite paths and corresponding signal carrier-to-noise (C/N0) ratios are plotted in Figure 13 and Figure 14. Good overall satellite reception (C/N0 values higher than 40 dBHz) are recorded in most areas of the skyplot. 

**Figure 15:**
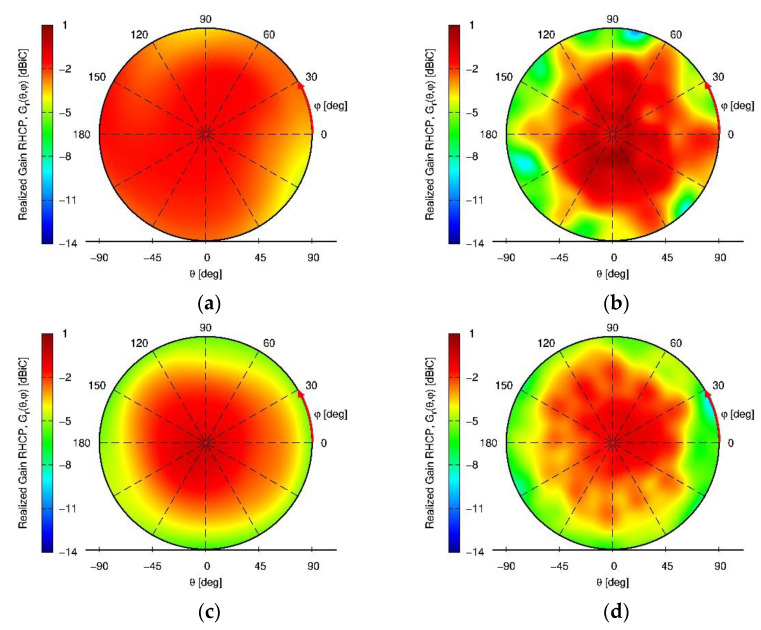
Measured RHCP and LHCP realized gain patterns of the miniaturized GNSS array without vertical choke rings standalone or installed on a hex-rotor UAV with installation height of 110 mm. (**a**) L5/E5a, standalone; (**b**) L5/E5a, on UAV h = 110 mm; (**c**) L1/E1 standalone; (**d**) L1/E1, on UAV h = 110 mm.

**Figure 16:**
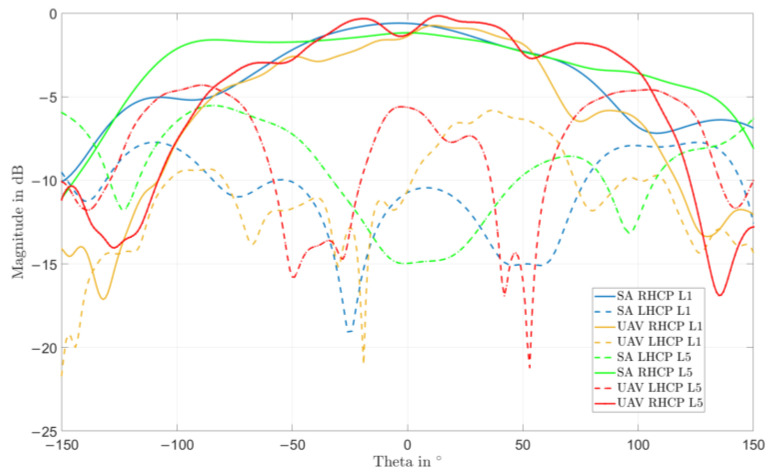
Phi = 0° cut of the measured RHCP and LHCP realized gain patterns of the miniaturized GNSS array (without vertical choke rings) standalone (“SA”) or installed on a hex-rotor UAV (“UAV”) with an installation height of 110 mm.

Very low elevations are experiencing lower C/N0 values once on UAV, due to azimuthal oscillations in the pattern at low elevations (as expected from the measured patterns shown in Figure 4 and Figure 10 and reported for easiness of reading in Figure 15 and (for a single cut) in Figure 16).

On the other side, the C/N0 values of the array installed on the UAV appear to have less oscillations over the entire satellite path, most notably at the L5/E5a band. This was not related to the antenna pattern itself and hence hints at possible multipath effects contributing to oscillations through destructive and constructive interference with line of sight signals. For the array installed on the platform, the effect is lower probably thanks to a good level of shielding provided by the platform itself against multipath from ground bounces. This is also confirmed by the patterns shown in Figure 16, where the cross-polarized component (LHCP) for directions close to nadir is about 5 dB higher at L5/E5a in standalone configuration than in the UAV case, and hence the antenna suppresses less ground multipath.

**Figure 17:**
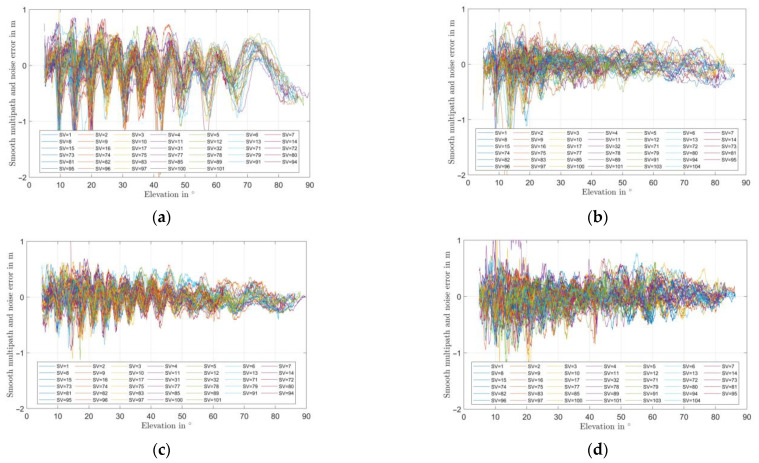
Estimated pseudorange multipath from GNSS measurements of the miniaturized GNSS array without vertical choke rings standalone or installed on a hex-rotor UAV with installation height of 110 mm. (**a**) L5/E5a, standalone; (**b**) L5/E5a, on UAV h = 110 mm; (**c**) L1/E1 standalone; (**d**) L1/E1, on UAV h = 110 mm.

In order to validate the last observation, 100 s smoothed pseudorange multipath errors have also been estimated from GNSS measurements through Code-minus-Carrier (CMC) techniques, as detailed e.g., in [16]. The results are shown in Figure 17. Though the multipath level at the L1/E1 band is slightly higher for the UAV case (mostly at low elevations, as explained before), at the L5/E5a band, the error is actually notably smaller when the array is installed on the UAV with respect to the standalone configuration, due to a beneficial shielding effect of the platform from ground multipath. This confirms the validity of the installation point as well as its usability on UAV for precise positioning.

## 5. Conclusions

This work has shown the development and test of a 2 × 2 GNSS antenna array, able to provide precise and robust navigation in GNSS-denied environments due to its phased array configuration. Miniaturization and weight reduction have been in focus as well as the impact of the installation of the array on the UAV platform on the overall patterns. Good radiation properties could be demonstrated by making use of the insight on the optimal placement won through installation performance analysis. The usability of the antenna on a real UAV platform has been demonstrated both in an anechoic chamber as well as in GNSS field tests.

## Data Availability

Not applicable.

## References

[1] Cui S., Chen Y., Li X. (2022). A Robust and Efficient UAV Path Planning Approach for Tracking Agile Targets in Complex Environments. Machines.

[2] Pinker A., Smith C. (1999). Vulnerability of the GPS Signal to Jamming. GPS Solut..

[3] Kerns A.J., Shepard D.P., Bhatti J.A., Humphreys T.E. (2014). Unmanned Aicraft Capture and Control via GPS Spoofing. J. Field Robot..

[4] Purisai S., Sharar O. (2022). A Robust Navigation Solution to Enable Safe Autonomous Aerospace Operations. SAE Tech. Pap..

[5] Basta N., Dreher A., Caizzone F., Sgammini M., Antreich F., Kappen G., Irteza S., Stephan R., Hein M.A., Schäfer E. System Concept of a Compact Multi-Antenna GNSS Receiver. Proceedings of the German Microwave Conference (GEMIC).

[6] Volakis J.L., O’Brien A.J., Chen C.-C. (2016). Small and Adaptive Antennas and Arrays for GNSS Applications. Proc. IEEE.

[7] Varma S., Caizzone S. Dual Band GNSS Antenna with High Back Lobe Suppression. Proceedings of the 2020 14th European Conference on Antennas and Propagation (EuCAP).

[8] Hehenberger S., Tripathi V., Varma S., Elmarissi W., Caizzone S. A Miniaturized All-GNSS Bands Antenna Array Incorporating Multipath Suppression for Robust Satellite Navigation on UAV Platforms. Proceedings of the 2021 15th European Conference on Antennas and Propagation (EuCAP).

[9] Caizzone S. (2016). Miniaturized E5a/E1 Antenna Array for Robust GNSS Navigation. IEEE Antennas Wirel. Prop. Lett..

[10] Caizzone S., Buchner G., Circiu M.-S., Cuntz M., Elmarissi W., Marcos E.P. (2019). A Miniaturized Multiband Antenna Array for Robust Navigation in Aerial Applications. Sensors.

[11] Caizzone S., Varma S., Buchner G., Elmarissi W. 3D Printed All-GNSS Bands Miniaturized Antenna and Array for Robust Satellite Navigation. Proceedings of the 2020 14th European Conference on Antennas and Propagation (EuCAP).

[12] https://catalog.ides.com/Datasheet.aspx?I=19843&FMT=PDF&E=438006&SKEY=19843.1484624.235739633%3A2e1bb653-c21c-476a-bc31-fce6405ec9e2&CULTURE=en-US&.

[13] Goulas A., Zhang S., Cadman D.A., Järveläinen J., Mylläri V., Whittow W.G., Vardaxoglou J.C., Engstrøm D.S. (2019). The Impact of 3D Printing Process Parameters on the Dielectric Properties of High Permittivity Composites. Designs.

[14] Abdalmalak K.A. (2022). Standing-Wave Feeding for High-Gain Linear Dielectric Resonator Antenna (DRA) Array. Sensors.

[15] Tamjid F., Foroughian F., Thomas C.M., Ghahreamani A., Kazemi R., Fathy A.E. (2020). Toward High-Performance Wideband GNSS Antennas-Design Tradeoffs and Development of Wideband Feed Network Structure. IEEE Trans. Antennas Propag..

[16] Caizzone S., Tripathi V., Hehenberger S. Investigating GNSS Multipath in Aeronautic Applications Through Antenna Installed Performance. Proceedings of the 2021 15th European Conference on Antennas and Propagation (EuCAP).

[17] Wang X., Li J., Chen W., Zhang M., Chen J., Zhang A. (2020). The Effect of Mutual Coupling on the Performance of GNSS Antenna Arrays. IEEE Access.

